# Genome modification in plant mitochondria

**DOI:** 10.1093/plphys/kiaf197

**Published:** 2025-05-22

**Authors:** Joachim Forner

**Affiliations:** Max-Planck-Institut fuer Molekulare Pflanzenphysiologie, Abteilung 3, Am Muehlenberg 1, Potsdam D-14476, Germany

## Abstract

Mitochondria are an indispensable component of every plant cell and are inextricably linked to many vital functions. One of their key characteristics is that they have their own genome. This genome, although greatly reduced, encodes several essential genes. While this has been known for decades, until recently it has not been possible to study the mitochondrial genome and its function in detail due to the lack of suitable tools for forward and reverse genetics. This is partly due to the low mutation rate in mitochondria and the lack of methods for direct transformation. A breakthrough came with the use of nuclear encoded transcription activator-like effector (TALE) nucleases (TALENs) for targeted mitochondrial mutagenesis. One of the first applications was to unambiguously show that certain ORFs were causal for cytoplasmic male sterility (CMS). This had previously been beyond our technical capabilities. TALENs are suitable for all plant species amenable to nuclear transformation because they are protein-only and can be imported post-transcriptionally into the mitochondria. Unfortunately, TALEN mutagenesis in plant mitochondria often seems to be associated with large genomic rearrangements. DNA base editors, the latest addition to the toolbox, bypass these side effects and merely introduce point mutations. They are based on TALEs and could only be developed after the discovery of a cytosine deaminase that acts on double-stranded DNA. The possibilities for targeted modification of the mitochondrial genome in plants are developing rapidly. This article aims to show where we stand in this development and what we can expect in the near future.

## Introduction

Mitochondria play a pivotal role in eukaryotic cells, including plant cells. They are key players in energy metabolism and also perform a number of other important functions (Moller et al. 2021; Monzel et al. 2023). They run the tricarboxylic acid cycle and generate ATP from its products via the oxidative phosphorylation pathway. Plant mitochondria also play an important role in photorespiration. Beyond that, to name but a few, they are involved in programmed cell death and the response to pathogens and host many anabolic reactions such as the biosynthesis of vitamin B9 and that of iron-sulfur centers. Descended from formerly free-living bacteria (Sagan 1967; Martin et al. 2015), mitochondria have retained their own genome—albeit with a greatly reduced gene content, nowhere near enough to encode all the proteins needed in mitochondria—and the machinery for its expression. These genomes contain only a few dozen genes, mostly encoding subunits of the respiratory chain for oxidative phosphorylation or components of the gene expression machinery such as ribosomal proteins or tRNAs (Unseld et al. 1997; Sugiyama et al. 2005). Research into plant mitochondria has long been hampered by the lack of suitable tools for modifying their genome. While true in planta transformation is still not yet possible, the breakthrough then came in 2019, when Kazama and co-workers (Kazama et al. 2019) reported the first higher plants (rice and rapeseed) with targeted mutations in their mitochondrial genomes. This was made possible by transcription activator-like effector nucleases (TALENs) (Boch et al. 2009; Christian et al. 2010). These protein-only, site-specific DNA endonucleases with easily programmable recognition specificities can be delivered as transgenes to the nucleus, and the resulting proteins can be delivered to the mitochondria simply by adding an N-terminal mitochondrial presequence. There they cause targeted DNA double-strand breaks, which are on occasion imperfectly repaired, resulting in mutant alleles of the target locus. Since then, a lot of things have happened in rapid succession. Several groups have reported the use of TALENs for targeted mutagenesis of mitochondrial genes in various plant species. On top of this, in the last 2 years, also TALE-based DNA base editors have been applied to modify plant mitochondrial genomes. They are based on the discovery of a cytosine deaminase that acts on double-stranded DNA, thus enabling targeted C-to-T conversions. By fusing an additional adenosine deaminase domain, even targeted A-to-G conversions are now possible. This article will elaborate on the recent achievements in the modification of plant mitochondrial genomes and provide an outlook on the imminent future progress to be expected.

## TALEN-mediated gene deletions

The major recent advances in plant mitochondrial genome engineering have been in the area of targeted gene deletion using TALENs. This review focuses on the application of TALENs, and readers interested in technical details are referred to other publications that cover these aspects more extensively. Thus, for an overview of the exact structure of the TALENs, what elements they consist of, the different types that are available, how they gain their specificity, how to clone them, and why they usually act in pairs, see for example (Binder et al. 2014; Ousterout and Gersbach 2016; Becker and Boch 2021; Tan et al. 2022). It is worth noting that TALENs can also be targeted to sites not preceded by a thymine residue (Lamb et al. 2013; Becker and Boch 2021).

As far as the practical implementation is concerned, the TALEN mutagenesis described below has usually been achieved using constitutive promoters. Depending on the species, this has been done in T0 or T1 plants, in tissue culture, or after seed passage in plants developing under regular conditions. Transformation has been carried out using *Agrobacterium tumefaciens*.

## Cytoplasmic male sterility (CMS) genes

CMS, the heritable inability to produce functional pollen due to alterations in the maternally inherited cytoplasmic (here mitochondrial) genomes is an intriguing biological feature with lucrative applications in hybrid seed production. Research on this topic has been going on for decades, but due to the lack of genome modification techniques, it has never been possible to rigorously prove that the identified mitochondrial candidate ORFs are indeed the cause of the observed CMS phenotype.

In their landmark 2019 paper (Kazama et al. 2019), Kazama and colleagues were the first to remove candidate genes for CMS from the mitochondrial genome, *orf79* from Boro-Taichung CMS rice, and *orf125* from Kosena-type CMS rapeseed. Male fertility was fully restored in the resulting plants, demonstrating that the 2 ORFs in question are indeed the cause of CMS. It is also worth noting that both the absence of the CMS ORF and the fertility phenotype persisted even when the offspring in the female line did not inherit the TALEN transgene, proving that the elimination of the wild-type genome had been complete and that there was no hidden substoichiometric heterochondriomy.

**Figure 1.**
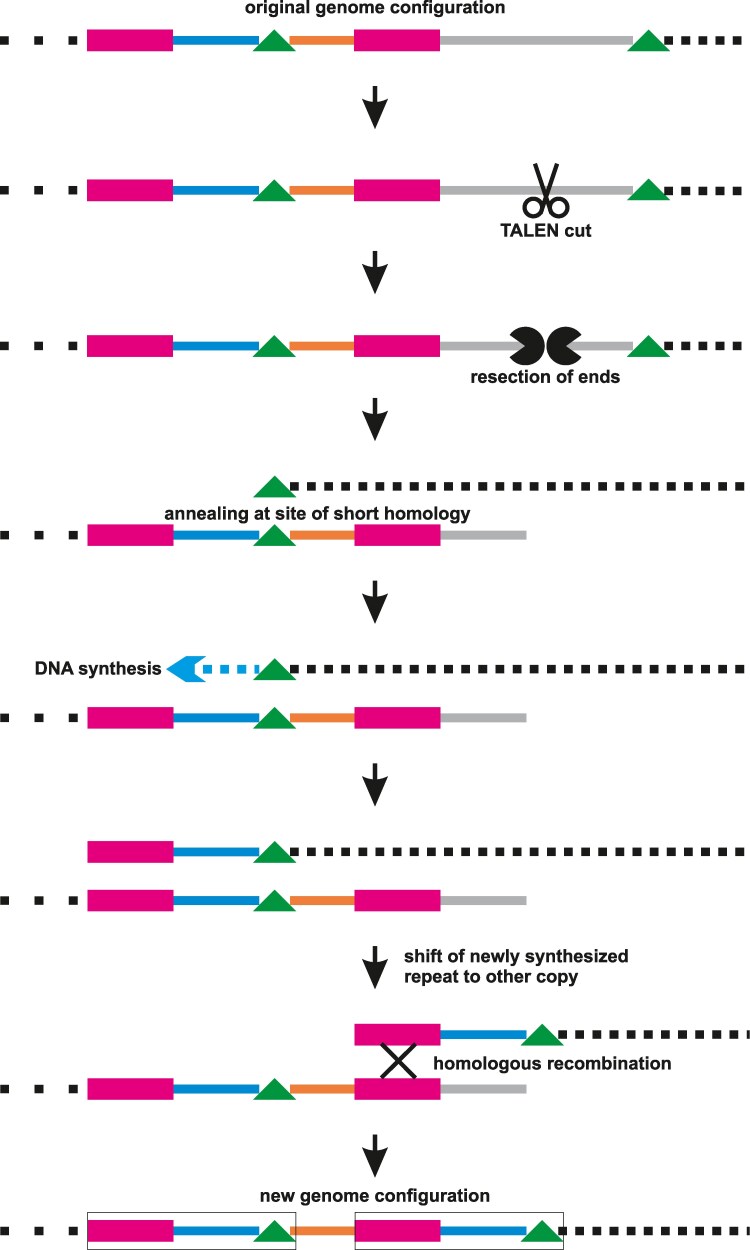
One possible way to repair cut mitochondrial genomes. Depicted are 3 unique sequences (cyan, orange, grey lines), a pair of long repeats (magenta boxes), and a pair of short repeats (green triangles); the rest of the genome extends on both sides (black dotted lines). Once the TALENs (black scissors) have made a double-strand break in the DNA, the open ends are resected at both sites. In the example given here, the sequence on the right of the cut is trimmed back until the short repeat sequence (green triangle) is reached. The exposed end is then aligned with the second copy of the short repeat by base pairing and DNA synthesis is initiated. Once the first long repeat (magenta) has been copied, the newly synthesized copy can undergo homologous recombination (HR) with the other original copy of the repeat, repairing the double-strand break. The net effect is to delete the unique sequence (grey), including the TALEN target site, and duplicate the unique sequence (cyan) between the first copies of the short and long repeats, resulting in an even larger repeat (framed in black). Note that in practice the extent of the deletion is limited by the next essential genes on either side, whereas the extent of the duplication is in principle unconstrained.

Knocking out genes in the nucleus with TALENs and also with CRISPR/Cas9 usually results in small insertion/deletion (indel) mutations, typically ranging from 1 nucleotide to several tens of nucleotides. This is the typical outcome of nonhomologous end-joining (NHEJ), the dominant repair pathway for DNA double-strand breaks in the plant nucleus. Incidentally, if the repair happens to be perfect, the event will never be visible. In plant mitochondria, there is no NHEJ, but broken molecules are repaired by homologous recombination–dependent repair using intact genome copies as repair templates (Gualberto and Newton 2017), a form of homologous recombination (HR). This process simply restores the original wild-type sequence. In rare cases, microhomologies are used to join the broken end to another part of the genome by microhomology-mediated end joining (MMEJ) (Garcia-Medel et al. 2019), which is mutagenic. When this is done using sequences on both sides of the break, a large deletion (often in the order of kilobase pairs) is created around the TALEN target site, leaving the rest of the genome unaltered. Very often, however, each side of the break is joined separately to distant sequences of the mitochondrial genome, resulting in large genome rearrangements— for example, a net deletion around the TALEN target site coupled with a duplication of other parts of the genome (see Fig. 1 and Box 1). Since there are many short stretches of sequence that could serve as putative microhomologies, the exact outcome of TALEN cuts in the mitochondrial genome is highly unpredictable. Another restriction is that the net deletion cannot include a gene essential for mitochondrial function, thus limiting the number of possible outcomes.

**Box 1.** Side effects of deleting mitochondrial genes via TALENs The inactivation of genes by TALEN-mediated targeted DNA double-strand breaks is often plagued by an unintended and undesirable side effect in plant mitochondria. While in the plant nucleus such an approach usually results in mutant alleles with a small insertion or deletion (indel) around the target site, the outcome in the mitochondrial genome is quite different. There, the deletions commonly observed do not affect 1 to several tens of nucleotides but usually span large sequences, typically hundreds or thousands of base pairs. The extent of these deletions is not predictable. To make matters worse, in most cases the sequences at the 2 borders of the large deletion are not simply stitched together but are instead individually fused to 2 different distant parts of the mitochondrial genome. Instead of a simple deletion allele, a rearranged mitochondrial genome is formed. The new genome configuration is likely to include a sequence duplication, creating a new large repeat in the mitochondrial genome in addition to the net deletion around the target site (Fig. 1). All these effects are highly undesirable as they complicate downstream analyses. These large net deletions are limited to the sequence between the closest adjacent essential genes on either side of the cut. This is not because larger deletions involving these essential genes would be mechanistically impossible but because cells in which the cut is repaired in this way are simply not viable, and the plants concerned will therefore never be recovered. While nothing can be done to control the outcome of repair at the nucleotide level, if enough plants are screened, it should be possible to recover at least the “clean” deletions without additional genomic changes. This unpredictability is due to the repair pathway employed in plant mitochondria. There is no NHEJ as in the nucleus and RDR using uncut genome copies as templates is probably the dominant repair pathway in mitochondria. Therefore, mutated alleles are recovered only in those few instances where MMEJ is used for DNA repair. After resection of the original DNA ends formed at the TALEN cut site, exposed short sequences that happen to have an identical copy elsewhere in the genome are used to join the loose DNA to the other part of the mitochondrial genome at the other copy of the short sequence. These resections can span hundreds to thousands of nucleotides, exposing many potential microhomologies, which explains the vast number of putative repair outcomes.

Since 2019, this technique has been very productive in unambiguously identifying additional CMS loci in different plant species. For example, *orf312* has been shown by TALEN-mediated deletion to be the causal gene for Tadukan-type CMS (TAA) in rice (Takatsuka et al. 2022) and *orf137* for the CMS phenotype in the tomato line Dwarf CMS[P] (Kuwabara et al. 2022). It has also been shown by TALEN-mediated knockout (Zhou et al. 2024b) that *WA352c* is the causal gene for CMS-WA in rice, a gene very similar to *orf352* of the rice RT102-type CMS gene, whose TALEN-induced deletion only partially restored male fertility (Omukai et al. 2021). In broccoli, deletion of *orf138* restored fertility to the Ogura-type CMS phenotype, but only at high temperatures, creating a temperature-switchable CMS system (Xu et al. 2024). Interestingly, also in *Arabidopsis thaliana*, a primarily selfing species, a TALEN-mediated knockout unambiguously identified *orf117Sha* as the causal gene for the gametophytic Sha-CMS (Dehaene et al. 2024).

## Regular genes

The use of TALENs for mitochondrial genome modification is not limited to CMS but has also been applied to evolutionarily conserved genes. The first was *atp6* in *A. thaliana*, or more precisely the 2 paralogs *atp6-1* and *atp6-2*, which encode a subunit of ATP synthase. Both could be deleted individually but not in combination, showing that *atp6* is an essential gene and that both copies are functional (Arimura et al. 2020). Also in *A. thaliana*, *nad7* (encoding a subunit of complex I of the respiratory chain) was targeted for mutagenesis by TALENs. In this case, however, it was not possible to obtain homochondriomic and viable plants. If the seeds germinated at all, the resulting plants died at the latest before bolting (Ayabe et al. 2023). Thus, at least in *A. thaliana*, *nad7* is an essential gene. In contrast, the loss of *nad9* (another subunit of complex I) is not lethal in *Nicotiana tabacum* (Forner et al. 2023). While homochondriomic plants showed a strong phenotype, namely delayed germination and growth, altered leaf morphology as well as male sterility, stable lines could be established that faithfully transmitted the mutation to their progeny. The phenotype could be fully complemented by allotopic expression of *nad9* as a nuclear transgene with a mitochondrial presequence targeting the resulting protein to the mitochondria. This is both a proof of concept for the creation of a fully artificial CMS system, with the nuclear copy acting as a restorer of fertility and the wild type as the maintainer line, and an example of artificial endosymbiotic gene transfer from the mitochondrion to the nucleus, a process that has been ongoing since the establishment of endosymbiosis. A similar experiment had previously been performed with *nad7* in *Nicotiana sylvestris* (Pineau et al. 2005), where, unlike in *A. thaliana*, *nad7* is not essential. But rather than being a technical breakthrough that served as a blueprint for similar future experiments, in this case the recovery of a *nad7* knockout line after protoplast culture was an unrepeatable stroke of luck (Pla et al. 1995), in the sense that protoplast culture as used there can occasionally induce rearrangements in the mitochondrial genome, but not in a predictable or controllable manner. As an aside, in the case of *N. tabacum nad9*, 3 out of 31 lines obtained showed clean deletion alleles—that is, no additional genome rearrangements—demonstrating that TALEN mutagenesis has the potential to produce knockouts with minimal side effects.

## Mutagenesis by DNA base editors

Very recently, the elegant concept of DNA base editing has been introduced into the field of plant mitochondrial genome modification. With this method, defined C (cytosine) residues in the genome can be changed to T (thymine) and, correspondingly, on the opposite DNA strand, G (guanine) to A (adenine). This breakthrough was made possible by the discovery and biotechnological adaptation of DddA (double-stranded DNA deaminase toxin A) from *Burkholderia cenocepacia*, a cytidine deaminase that acts on double-stranded DNA (Mok et al. 2020). Before this, DNA base editors used for genome modification were based on a fusion of catalytically impaired Cas9 and cytidine deaminases derived from RNA editing (Komor et al. 2016; Nishida et al. 2016). These cytidine deaminases normally act on single-stranded RNA, and the derived versions used in genome engineering still act on single-stranded targets, in this case DNA. When the Cas9 protein/sgRNA complex binds its target region in the genome, 1 strand of the target sequence is necessarily exposed as a single strand, since the protospacer region of the sgRNA forms an RNA-DNA duplex with the other strand, allowing cytosine deamination. This requirement for single-stranded DNA regions has precluded the use of DNA base editors in plant mitochondria, since CRISPR/Cas9 cannot (yet) be used for mitochondria (because sgRNAs cannot be delivered) (Schmiderer et al. 2022, see Box 2) and TALE-based approaches do not generate single-stranded DNA regions. With DddA, however, double-stranded DNA can now be used as a template for cytosine deamination, fused to a TALE backbone for specific DNA binding (Mok et al. 2020). Only the C-terminal deamination domain of DddA is required for cytosine deamination, but even when fused to a TALE backbone, it would unspecifically deaminate Cs throughout the genome. For this reason, it was split into 2 parts, each catalytically inactive on its own and each part fused separately to a TALE backbone. Only when the 2 halves come into close spatial proximity upon binding of the target region is a functional deaminase restored and Cs in the target region can be deaminated, a setup similar to TALENs with their 2 FokI domains. It was named DddA-derived cytosine base editor (DdCBE). The direct product of cytosine deamination is uracil, not thymine—uracil simply behaves like thymine in base pairing, leading to the incorporation of adenine into the opposite strand during DNA replication. As uracil is removed from DNA during base excision repair by Uracil-DNA glycosylase, a third component, Uracil-DNA glycosylase inhibitor, is added to DdCBEs to prevent Uracil-DNA glycosylase from acting and thus increase the chances of the base change being stably incorporated into the genome.

**Box 2.** Why TALENs and not CRISPR/Cas? Mitochondrial transformation in planta is not yet possible, so we can only modify the mitochondrial genome with components that are imported into the mitochondria from the cytoplasm. In general, this is done by transforming the nucleus. The nuclear transgenes encoding the editing components are then expressed, and the resulting gene products are delivered to the mitochondria. This is relatively easy to achieve with TALENs and other TALE-derived genome editing tools, as they are protein-only, and importing proteins encoded by nuclear transgenes from the cytoplasm by simply adding an N-terminal mitochondrial presequence has been standard practice for decades (Logan and Leaver 2000). However, CRISPR/Cas9-based genome editing tools not only consist of proteins but also require an essential RNA component (Doudna and Charpentier 2014). And unfortunately, the import of transgene-derived RNA molecules from the cytoplasm into the mitochondria is a major limitation in mitochondrial genome engineering (Schmiderer et al. 2022). While for a long time it has been known that functional small RNA molecules, for example, ribozymes (Val et al. 2011), can be successfully imported piggy-backing on tRNAs—or more precisely mimics thereof—naturally imported from the cytoplasm (Salinas et al. 2008), there have been no reports of this concept being successfully applied to sgRNAs. Thus, for the foreseeable future, CRISPR/Cas-based techniques will not be available to modify plant mitochondrial genomes.

While the original publication described the use of DdCBEs in mitochondria of human cells in cell culture, it has also been successfully employed in plant mitochondria. The first report (Kang et al. 2021) presented the application in mitochondria from lettuce and rapeseed protoplasts, attempting to introduce a stop codon into the *atp6* gene using transient transformation with plasmids encoding the base editor. Up to 23% of the sequencing reads showed editing (i.e. C-to-T or G-to-A conversions) in the target window. However, these 23% were composed of mutations of the target C alone, mutations at other Cs or Gs in the target window without the intended C-to-T change, and combinations thereof. Such bystander edits are a common phenomenon in genome editing. Similarly, 11% of edited reads were found in rapeseed protoplasts transfected with plasmids encoding a DdCBE targeting the mitochondrial *rps14* gene. When measured 4 weeks after transfection in calli regenerated from transfected rapeseed protoplasts, base edits were detected at frequencies of up to 25% (for *atp6*) and 1.9% (for *rps14*), respectively, but only some calli showed targeted mitochondrial base edits. However, no stable plant lines with base-edited mitochondria were established.

The same concept has also been applied to *A. thaliana* (Nakazato et al. 2022), naming their base editor mitoTALECD. In this case, stable transgenic plant lines were generated after nuclear floral dip transformation. In some cases, the induced mutations in the mitochondrial genome were faithfully transmitted to the next generation, even when the transgene encoding the base editor was not co-transmitted and all copies of the mitochondrial genome were mutated (i.e. homochondriomy was achieved). This means that all the criteria for establishing a mutagenesis pipeline were met. The target nucleotide was C1178 in the *atp1* gene. In the wild type, this C is edited at the RNA level, changing codon 393 from serine to leucine. This RNA editing requires the PPR protein OTP87, and plants lacking OTP87 and therefore lacking the editing in the *atp1* mRNA are visibly impaired. As expected, DNA base editing of C1178 rescued the OTP87 knockout phenotype. Some plants containing only the desired mutation (no bystander mutations) were obtained. The authors also found no off-target mutations in other regions of the mitochondrial genome, underscoring the usefulness of base editors. Since the original discovery of BcDddA (from *B. cenocepacia*), the tool set has been greatly expanded (excellently reviewed in (Tang and Du 2025)). Both engineering by mutagenesis and identification of DddA orthologs from other species have allowed problems such as the strong preference for 5′-TC, bystander mutations (i.e. the width of the editing window), and the lack of strand specificity to be overcome. They also increased editing efficiency and reduced off-target activity. However, these improved versions have not yet been tested in plants.

For details on the general design, mode of action, and cloning of (mitochondrial) base editors, see, for example (Mok et al. 2020; Becker and Boch 2021; Tan et al. 2022; Nakazato and Arimura 2025).

The scope of mitochondrial DNA base editing is not limited to C-to-T but has recently been extended to A-to-G editing (including T-to-C changes when considering the opposite strand). Again, as with C-to-T editing, this is based on work with the CRISPR/Cas9 system and was first demonstrated in human mitochondria (Cho et al. 2022). In essence, it is an extension of the DdCBE setup. The key enzyme here is TadA8e, a modified form of *Escherichia coli* tRNA adenosine deaminase (TadA), which is involved in tRNA maturation and deaminates adenosine residues. It produces inosine as an intermediate, which behaves like guanosine in base pairing, causing the incorporation of a cytosine into the opposite strand during DNA replication. The DddA component is still required to make the target DNA accessible (i.e. single-stranded) but it can be catalytically inactive with regard to cytosine deamination. When both TadA8e and DddA are functional, it is a dual editor that can simultaneously edit both Cs and As in the target window. When first applied to plant mitochondria (Zhou et al. 2024a), the *nad7* and *atp6-2* genes of *A. thaliana* were targeted. And indeed, the authors were able to provide proof of concept. In the progeny of a single T1 plant, they analyzed 28 T2 plants that had not inherited the gene for the base editor, and 14 out of these were homochondriomic for an A-to-G substitution in the *atp6-2* target window.

However, there appears to be much room for optimization as only 1 of the 4 constructs tested yielded such plants; none were reported for the other *atp6-2* and the 2 *nad7* constructs. A monomeric TALE-linked adenine deaminase with a catalytically dead DddA domain under the control of the *RPS5A* promoter was used in this study. With recent improvements in adenosine base editors (Tang and Du 2025), progress toward more efficient A-to-G editing in plant mitochondria is expected in the near future.

## Nucleases and deaminases in comparison

As the field is still in its infancy and evolving rapidly, it is certainly too early to provide guidelines and general recommendations on which of the available tools should be used for a given mutagenesis project. Nevertheless, there is already a case study in which the authors systematically compared the inactivation of a mitochondrial gene by 2 approaches, namely TALENs (mitoTALENs) and cytosine base editors (mitoTALECDs) (Nicolia et al. 2024). Their target gene was *orf125* from potato, a candidate for CMS. They tested 2 constructs each for TALENs and TALECDs. In brief, they were successful in all cases. For the TALENs, they obtained deletion alleles in 3 out of 15 transgenic plants tested for construct #1 and in 8 out of 14 instances for construct #2. Of these positive cases, 0 and 4 were homochondriomic. As for the base editor, the desired stop codons were introduced in 12 out of 21 plants for construct #1 and in 10 out of 22 plants for construct #2. Of these, 1 and 8, respectively, were homochondriomic. Most importantly, in all cases there was no reversion to wild type during vegetative propagation for the homochondriomic knockout mutations. In addition to efficacy, there are other parameters to consider. The base editors created additional by-stander mutations, on the same genome molecules as the intended stop codons. For the purpose of creating knockout alleles, however, this is not really a concern. And in this particular example, all the knockout alleles obtained using TALENs were clean deletions of 236 bp or 1066 bp in the sense that no additional genome recombination was involved. This seems somewhat atypical but might be explained by the fact that: a) an important gene (*nad4*) was close by; and b) that 2 sets of small direct repeats were available in close proximity for ligation of both ends of the cut. Persistent heterochondriomy was also observed in both TALENs and TALECDs during vegetative propagation, suggesting that both are equally susceptible to rather early transgene silencing. As the authors of the manuscript under discussion only focused on the technical aspects and did not investigate the intended phenotype—that is, reversion to male fertility, the efficiency in achieving the actual goal cannot be assessed and compared yet.

## TALEN gene-drive mutagenesis (TALEN-GDM)

The TALEN-GDM method (Forner et al. 2022) is somewhat unique among the mitochondrial genome modification approaches reported here. It differs from the others in several key aspects. First, even though the main outcome of TALEN-GDM is the substitution of single nucleotides, it does not directly modify nucleotides in the genome, in contrast to base editors. Second, although it also is based on cuts in the genome like standard TALEN mutagenesis for gene deletion, it does not rely on the erroneous repair of DNA double-strand breaks. Third, it selects for mutations in the binding sites of the TALE repeats, not adjacent to them. Fourth, there is a strong element of chance involved in the exact nature of the resulting mutation. And, in particular, it is absolutely dependent on the selection pressure that is exerted directly on the DNA—a feature it shares, incidentally, with TALEN-induced gene deletion, although it is not an essential component of the latter approach.

All the techniques presented here, with the exception of the future concept of true transformation, share an element of chance in that the outcome is not fully predictable for all of them. However, the extent to which chance plays a role varies considerably. For base editors, whether they are based on cytosine or adenosine deaminases, the uncertainty about the outcome lies only in the rather wide editing window. Any accessible nucleotide within it can be edited or not in any conceivable combination. These combinations include, of course, the most common outcome of no change at all, as with all attempts at genome modification. However, the number of possible new alleles is rather limited. When nucleases (TALENs) are used, the main result is always the same: a complete deletion of the target locus. What is different each time is the configuration of the mutated genome. In some cases, the 2 open ends of the resected DNA can be directly joined together (via MMEJ or HR), while in others each end is individually joined to another homologous sequence of the genome, resulting in large deletions, partial duplications and possibly structural rearrangements.

In contrast, TALEN-GDM, like base editors, does not induce structural changes. However, unlike base editors, TALEN-GDM is not limited to 2 types of transition mutations (C-to-T and A-to-G; which is G-to-A and T-to-C on the opposite strand). In principle, TALEN-GDM can create all possible transition (A-to-G, G-to-A, C-to-T, and T-to-C) and transversion (A-to-C, A-to-T, C-to-A, C-to-G, G-to-C, G-to-T, T-to-A, and T-to-G) mutations, as well as combinations thereof (as shown by the example of CG to AA in (Forner et al. 2022 )). It is also conceivable that small insertions or deletions spanning 1 to several nucleotides could be obtained by this method. Admittedly, this has never been demonstrated, but the number of isolated point mutants has never been nearly enough to saturate the mutational space. Notably, TALEN-GDM does not appear to induce point mutations in other parts of the mitochondrial genome, even when mutagens (ethidium bromide, N-ethyl-N-nitrosourea) are used.

**Figure 2.**
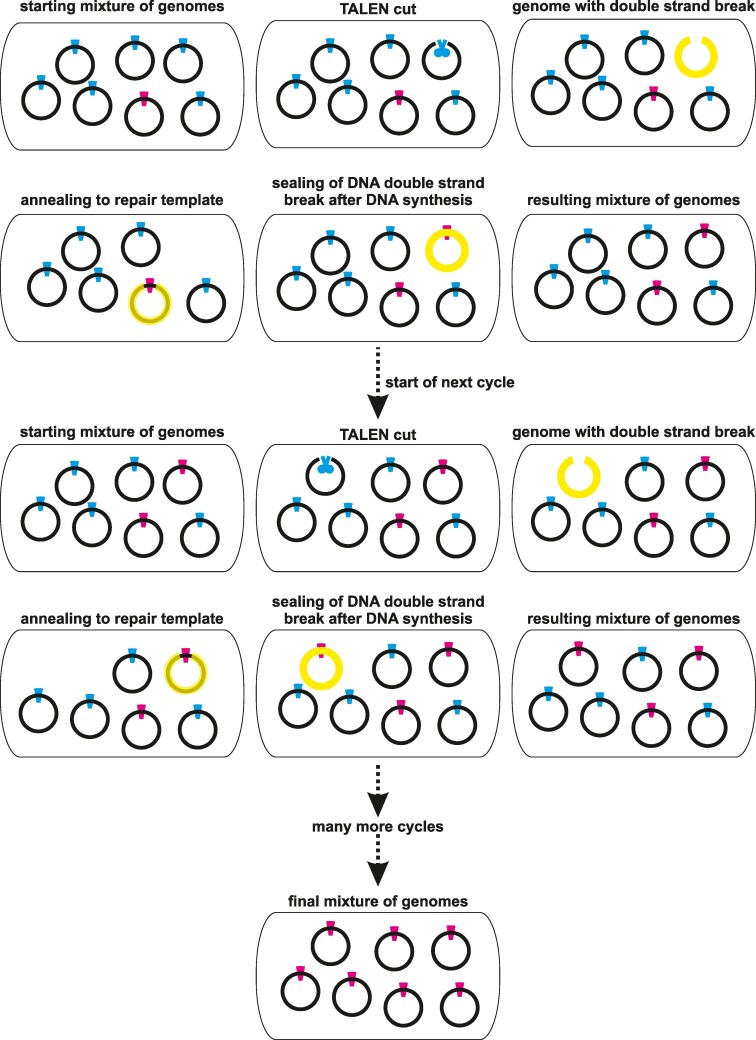
The concept of gene drive in the context of mitochondrial genome editing. Mitochondria are highly polyploid in the sense that a given cell (rounded rectangle) contains many copies of the mitochondrial genome (small circles), although a given individual mitochondrion may not contain a genome at all. In the presence of a sequence-specific DNA endonuclease (e.g. TALENs, shown here as cyan scissors), genomes are cut and repaired for example by recombination-dependent replication (RDR), using an uncut genome as a template for DNA synthesis. If 2 different variants of the genome coexist in the same cell (heterochondriomy) and 1 of the variants has a mutation (magenta) in the target site (cyan) of the endonuclease—for example, caused by a TALEN-induced deletion as shown in Fig. 1—the mutant form will not be cut or at least will be cut less frequently than the wild-type version. Since only intact (i.e. noncut) genome molecules can serve as a template for RDR repair of the cut genome (marked in yellow; superimposed on an intact genome serving as a repair template), it is possible that a cut genome is converted to the mutant form during repair. The opposite conversion, from mutant to wild type, is not possible (if the endonuclease target site is missing) or at least much less likely (if the affinity of the endonuclease for the mutated cut site is reduced, but not to zero). This results in an intracellular genetic sweep in which all wild-type alleles are rapidly converted to the mutant form by the presence of the sequence-specific DNA endonuclease alone, not by any external selection pressure. Note: The mitochondrial genomes are represented as monogenomic circles, but this is not necessarily how they exist in nature (Cheng et al. 2017).

How does TALEN-GDM work in detail? To understand this, it is important to remember some basic facts about plant mitochondria (see also Box 3). The mitochondrial genome is highly polyploid. This means that there are about 100 copies of the mitochondrial genome in each cell (Preuten et al. 2010). There may be more individual mitochondria per cell than copies of the genome, but since mitochondria are constantly fusing and splitting, they can easily exchange components, including copies of their genomes. In the presence of nuclear-encoded TALEN proteins, the mitochondrial genomes are continuously cleaved at the TALEN target site. Now, TALEN-GDM can only work if the TALEN activity is low enough to keep at least some of the genomes intact at all times but high enough to occasionally cleave at least some of the genomes. The cut genome molecules are then repaired by homologous recombination–dependent repair (Gualberto and Newton 2017) or amplified by recombination-dependent replication (RDR) (Morley and Nielsen 2017; Brieba 2019), using the intact molecules as a template. As long as there is no mutation in the TALEN binding site(s), there will simply be an equilibrium between cut and repair, resulting in no sequence change and only a certain percentage of genomic molecules remaining in a broken (i.e. cut) state. However, if a mutation occurs in the TALEN binding site by any mechanism (spontaneous mutation, mutagen-induced mutation, polymerase-induced mutation during repair), such a genome will be cut less frequently than the original wild-type genomes. This means that the ratio of cut to uncut genomes is lower than for the wild-type genomes. Accordingly, among the intact genomes that can serve as repair templates, the percentage of mutant genomes is higher than their overall percentage in the cell. Conversely, among the cut genomes that need to be repaired, the proportion of wild-type genomes is higher than their total proportion in the cell. Taken together, this means that wild-type genomes are more likely to be converted into mutant genomes during RDR-mediated repair than vice versa (see also Fig. 2). Thus, in a given cell, over time, all wild-type genomes—that is, those without a mutation in their TALEN binding site(s)—will be converted to the mutant form, with a mutation in their TALEN binding site(s). In other words, in the presence of the TALEN protein(s), the mutant genome has a selective advantage over the wild-type genome that is due only to the nucleotide sequence per se at the DNA level and not to any phenotype that it encodes. This constitutes a gene drive in which one allele outcompetes another during transmission to daughter cells. Classical gene drives (Courret et al. 2019) act at the level of gametes, whereas the one described here acts during mitosis and even in nondividing cells. Once the mutant genome has completely taken over the chondriome, a new equilibrium is reached where, at any given time, a lower percentage of mitochondrial genomes are cleaved than before. This will remain stable until an additional mutation occurs in the TALEN binding site(s), but this time the drive is likely to be weaker because the “old” alleles are already being cut less efficiently. The endpoint would be an allele where the TALEN proteins cannot bind at all. Incidentally, this is also true for regular TALEN mutagenesis. In this case, the TALEN binding site(s) are completely deleted, making the resulting new genome configuration completely resistant to further TALEN cuts. This effect does not occur with base editors, which explains why heterochondriomy (i.e. partial editing of the genome) is a problem for base editors, whereas TALEN-based systems are very quickly driven toward homochondriomy (at least at the intracellular level). Should 2 mutated alleles arise concomitantly in the same cell, the one that is cut less efficiently should prevail; if both are fully cut resistant, segregation is expected to occur either by pure chance or according to the phenotype caused by the alleles.

**Box 3.** Key features of plant mitochondrial genomes Plant mitochondrial genomes contain only a few dozen genes (e.g. 57 in *A. thaliana*). In essence, they encode the building blocks of the respiratory chain, either directly or indirectly via components of the mitoribosomes. The latter are, in turn, necessary to translate the former. The actual genes represent only a small fraction of the total genome sequence, the vast majority consisting of intergenic regions with no known function. The coding sequences are conserved to an extraordinary degree between species, whereas both the sequence and the structure of the intergenic region vary greatly. The mitochondrial genome also does not necessarily exist as a monogenomic circular molecule but in more complex structures of unclear stoichiometry. While an individual cell contains many copies of the mitochondrial genome and is thus highly polyploid, there may be more mitochondria than genome copies within a cell, so that at least some mitochondria do not contain the full complement of the mitochondrial DNA or even no DNA at all. This is alleviated by the constant fusion and fission processes in which the individual mitochondria equilibrate their contents.

All the mitochondria (or mitochondrial genomes in this context) within a given cell as a whole are referred to as the “chondriome.” If there is no allelic variation and the chondriome is therefore uniform, the cell is called “homochondriomic.” Otherwise, if at least 2 different alleles of a locus coexist, the cell is said to be “heterochondriomic.” “Homoplasmic” cells (with a uniform cytoplasm) are always “homochondriomic,” but “homochondriomic” cells can also be “heteroplasmic” because they might be “heteroplastomic” (i.e. have in-plastid variation).

For future applications, the principle of TALEN-GDM could be combined with base editors as mutagens. Not only would homochondriomy be achieved more quickly by using a TALEN directed against the unaltered wild-type sequence at the target site of the base editor, but additional TALEN proteins designed to bind alleles resulting from unintended by-stander mutations would help to recover only alleles with the intended edit. For this purpose, and probably in general, compactTALENs, which require only 1 TALEN arm, would be ideal, which would also coincidentally reduce the number of transgenes required, thus saving work and reducing the risk of gene silencing. In any case, it is unclear whether TALEN-GDM could work with a regular TALEN pair, as the reported TALEN-GDM results are based on an unintended 1-arm approach. The question with a 2-arm approach is whether a single mutation in a single binding site could have a large enough effect to reduce the overall efficiency of TALEN cutting to the point where a gene drive effect is triggered. It is conceivable that cutting with both arms will always be so efficient that the sweet spot of expression—where there is detectable cutting activity but not so much that genomes are always completely cut—can never be reached. Furthermore, it remains to be determined whether the increased recovery of mutant alleles by the use of ethidium bromide is due to a putative mutagenic activity or simply to a reduction in the number of genomes (Warren et al. 2017), creating a bottleneck effect.

Lastly, it would be useful if TALEN-GDM could be transferred to other species. To date, TALEN-GDM has only been used in tobacco (*N. tabacum*). In this plant, TALEN-GDM relies on repeated cycles of regeneration from leaf explants in tissue culture, ensuring passage through a single cell, using the CaMV 35S promoter. To transfer this technique directly to other plants, they must be amenable to tissue culture. However, the *A. thaliana* accession Columbia, for example, is not suitable for tissue culture. Thus, it would be necessary to identify promoters that confer the required level of expression either in the shoot apical meristem or in the egg cell.

## A brief note on the potential for spread and use in plant breeding

The term “gene drive” in TALEN-GDM may evoke associations with uncontrolled spread in (wild) populations (Noble et al. 2018). However, a key feature of TALEN-GDM is that it works at the level of individual cells, not at the population level. For the latter to occur, 2 conditions would have to be met. First, the nuclease gene, which is encoded in the nucleus and does not itself benefit from the drive effect, would have to spread through the population by some other mechanism. Second, paternal leakage (McCauley 2013)—breaking the usual uniparental maternal mode of inheritance of mitochondrial genomes that predominates in most higher plants, including tobacco (Svab and Maliga 2007)—would have to occur frequently. Only then would wild-type and mutant alleles meet in the same cell, giving the latter a chance to displace the wild-type alleles. So there is a double safeguard: The nuclease is not encoded at the site where it cuts, but even in a genome that is inherited in a different way. This is particularly true in the case of putative horizontal transmission between species (Bergthorsson et al. 2003). Both the mitochondrial and nuclear genomes would have to cross the species barrier and then come together in the same plant, while in the meantime either component alone would not confer a selective advantage. Nevertheless, gene drive or not, it should go without saying that if mitochondrially engineered plants are to be used in agriculture, any genome editing tool located in the nucleus should, as a precautionary measure, be outcrossed by default before the variety is released. Even if only to prevent the introduction of additional off-target mutations by a continuously active nuclease/deaminase. In the case of nonsexually propagated species such as potato, the nuclear gene could be removed using the Cre/lox system (Cuellar et al. 2006), for example. In fact, because of uniparental (maternal) inheritance, modification of the organelle genome can be considered an additional level of safety to ensure transgene containment compared with alteration of the nuclear genome (Daniell 2007). None of the mitochondrial genome changes described here should confer a selective advantage over wild-type plants in the field. This includes the knockout alleles of *nad7* and *nad9*, although these are CMS genes. While it is hypothesized that CMS alleles could spread through a population by conferring a reproductive advantage specifically to the female line (Fujii et al. 2011), in this case they would not do so due to the associated vegetative growth penalty.

Conceptually, targeted mutations introduced into the mitochondrial genome using the new genome editing tools are identical to those in the nucleus. The main practical difference is the uniparental mode of inheritance and therefore the lack of recombination via sexual reproduction. For the potential of using the newly available mitochondrial genome editing tools to improve agriculturally relevant traits, until transgene integration is within reach, the most promising route in the near future will be to try to create CMS genes without an associated vegetative phenotype in crop species where none currently exist.

## Imminent future progress to be expected

There are a number of incremental technical improvements on the horizon that will almost certainly be successfully applied to plant mitochondria in the near future. This can be said with confidence because, over the past few years, essentially all the techniques that have been shown to work for editing the genome in, for example, mammalian mitochondria or plastids, or both, ended up working in plant mitochondria (see e.g. the cytosine and adenosine base editors mentioned above). And much work is being done to improve mitochondrial base editors, as described above.

Future mitochondrial base editors that omit DddA are already being developed. First tested in rice protoplasts for nuclear and plastid genome editing, cytidine deaminase-exonuclease-nickase-TALE was shown to work in mitochondria of human HEK293T cell lines (Hu et al. 2024). A TALEN nickase cuts only 1 strand at the target site, then an exonuclease removes several bases from 1 strand starting from the nick, thus creating a small stretch of single-stranded DNA on which a “classical” ssDNA-specific cytidine deaminase can act. In contrast to DddA-based systems, this limits base editing to just 1 strand, thereby reducing the number of possible by-stander mutations. Initially developed in a modular fashion with each component expressed individually, the authors have also shown that this can be achieved as a fusion protein. A possible extension of this is AdDENT (Hu et al. 2024), which uses an adenosine deaminase but follows the same principle.

An adenine transversion editor, ACBE (Chen et al. 2024), is one of the latest examples of a whole suite of genome editing tools that are based on the main concept of creating an abasic site. In brief, this is essentially a slight modification of the concept of conventional DNA base editors. Here now, when a cytosine or adenosine residue is deaminated by the deaminase domain of the base editor, a corresponding glycosylase—fused to the base editor—removes the modified base but leaves the sugar phosphate backbone intact. In other words, an abasic site is created. The error-prone repair of such a site then opens up the possibility of also obtaining transversion mutations. If this can be installed on a TALE backbone instead of CRISPR/Cas9, it should also be applicable in plant mitochondria.

On a more speculative note, and in case of success representing a true qualitative improvement, it is tempting to hope that the attempts at applying the CRISPR/Cas9 technology also to plant mitochondria will be fruitful in the end. The primary challenge resides in the delivery of sgRNAs to the mitochondria (see Text Box 2). Although there are reports of successful application of CRISPR/Cas in mitochondria (Jo et al. 2015; Nikitchina et al. 2024) (and references therein), these findings have not been replicated. It is therefore to be hoped that either sgRNAs can be translocated into mitochondria via the VDAC channel (Salinas et al. 2006) using tRNA-like structures attached to them, or that co-importation with the Cas9 protein via TOM-TIM (Translocator Outer Membrane and Translocator Inner Membrane) proteins (Moller et al. 2021) is possible. Regardless of the mechanism, the development of a reliable method of sgRNA delivery to mitochondria would open up a whole field of possibilities. When compared with TALE-based systems, CRISPR/Cas9 systems are significantly more straightforward to engineer, as only the small protospacer encoding part of the sgRNA needs to be exchanged to modify specificity and not the entire array of 34 amino acid protein repeats. Moreover, RNA-sequence based approaches, such as prime editing (Anzalone et al. 2019), would be a viable option. Until now, we have only been able to delete defined mitochondrial genes with no control over the extent of the deletion, and to make targeted C-to-T and A-to-G edits, while TALEN-GDM enables a broader but less defined set of base changes. But with prime editing, we could rewrite small stretches of sequence at will, including insertions, which in the absence of true transformation is the closest we can get to transgene insertion. However, these approaches are still in their infancy, and it remains to be seen whether it will be possible to apply them routinely on a large scale in time or whether a breakthrough in true mitochondrial transformation will make them obsolete before they are fully developed. See also Outstanding questions box.

## Concluding remarks—mitochondrial transformation

If true transgenesis could be achieved, this would be a gamechanger in the field of mitochondrial genome engineering in plants. Certainly, all of the newly developed techniques discussed above would still be invaluable, especially since mitochondrial transformation would most likely have to be carried out by biolistics or similar methods. This would be much more labor-intensive and time-consuming than *Agrobacterium*-mediated transformation, the standard for nuclear transgenesis, and would even be impossible in certain species or cultivars, such as *A. thaliana accession* Columbia, which is recalcitrant to tissue culture. Nevertheless, as homologous recombination is active there, a reliable transformation procedure for plant mitochondria would allow full control over the mitochondrial genome, even to the point of complete replacement by a fully artificially designed sequence in the long term. This not only would increase the scope of basic research questions that can be addressed (such as the effects of intron removal or elimination of all editing sites) but especially open the gates to biotechnological applications like creation of new CMS systems or the production of valuable compounds. Despite some encouraging recent reports (Law et al. 2022), the available data do not allow to predict when this technique will finally be available.

Advances box

- Genome editing in plant mitochondria has made a great leap forward in the last decade: targeted mutations can now be induced.
- Transgenes encoding DNA-modifying (protein-only) enzymes are delivered to the nucleus, and the translated proteins are imported into the mitochondria.
- DNA double-strand breaks are created using TALENs. When repair does not happen faithfully, sequences on either side of the cut are joined to other parts of the genome (via MMEJ or HR), resulting in large net deletions around the target site. Usually this also causes genome rearrangements, but in rare cases the sequences on both sides are ligated to one another, creating a “clean” deletion. In any case, the exact outcome is unpredictable.
- TALE-based DNA base editors can induce targeted C-to-T and A-to-G mutations without genome rearrangements but with the risk of bystander mutations.

Outstanding questions box

- Why have we not yet been able to stably transform plant mitochondria? So far, mitochondrial transformation is only possible in yeast (*Saccharomyces cerevisiae*) and *Chlamydomonas reinhardtii*. Both species are unicellular and can live without an intact respiratory chain, and respective mutant strains are available. There, mitochondrial transformants can be selected by complementation of these mutations under conditions where growth is only possible with proper respiration. This cannot be transferred to plants, as such mutants are not obtainable. So, is it simply a question of finding a suitable selection system for plants? The reason is unlikely to be DNA delivery, as yeast and Chlamydomonas mitochondria are transformed using a gene gun (biolistic transformation), a method which works well also for plant plastid transformation.
- Failing that/to bridge the time until true transformation: Can nuclear-expressed CRISPR/Cas9-based methods be established in plant mitochondria? This would enable especially prime editing in plant mitochondria, that is, rewriting small stretches of the genome at will. However, this would require the routine delivery of (sg)RNAs to the mitochondria, a task that has not yet been solved satisfactorily.

## Acknowledgments

I would like to thank Kin Pan Chung for embarking on the journey of writing a review at the same time, thus providing me with the final nudge of motivation. Also, I would like to express my appreciation for the input of the two reviewers. I am especially grateful for the extraordinary dedication of reviewer #2, whose insightful comments really helped to improve the manuscript a lot.

## Data Availability

There are no new data associated with this article.
